# Supplementary material for: Establishment and Characterization of Free-Floating 3D Macrophage Programming Model in the Presence of Cancer Cell Spheroids
Source: Int J Mol Sci. 2023 Jun 28;24(13):10763. doi: 10.3390/ijms241310763 (PMC10341749; doi:10.3390/ijms241310763)
Supplement: Supplementary file 1 [file ijms-24-10763-s001.zip › ijms-2448250-supplementary.pdf]

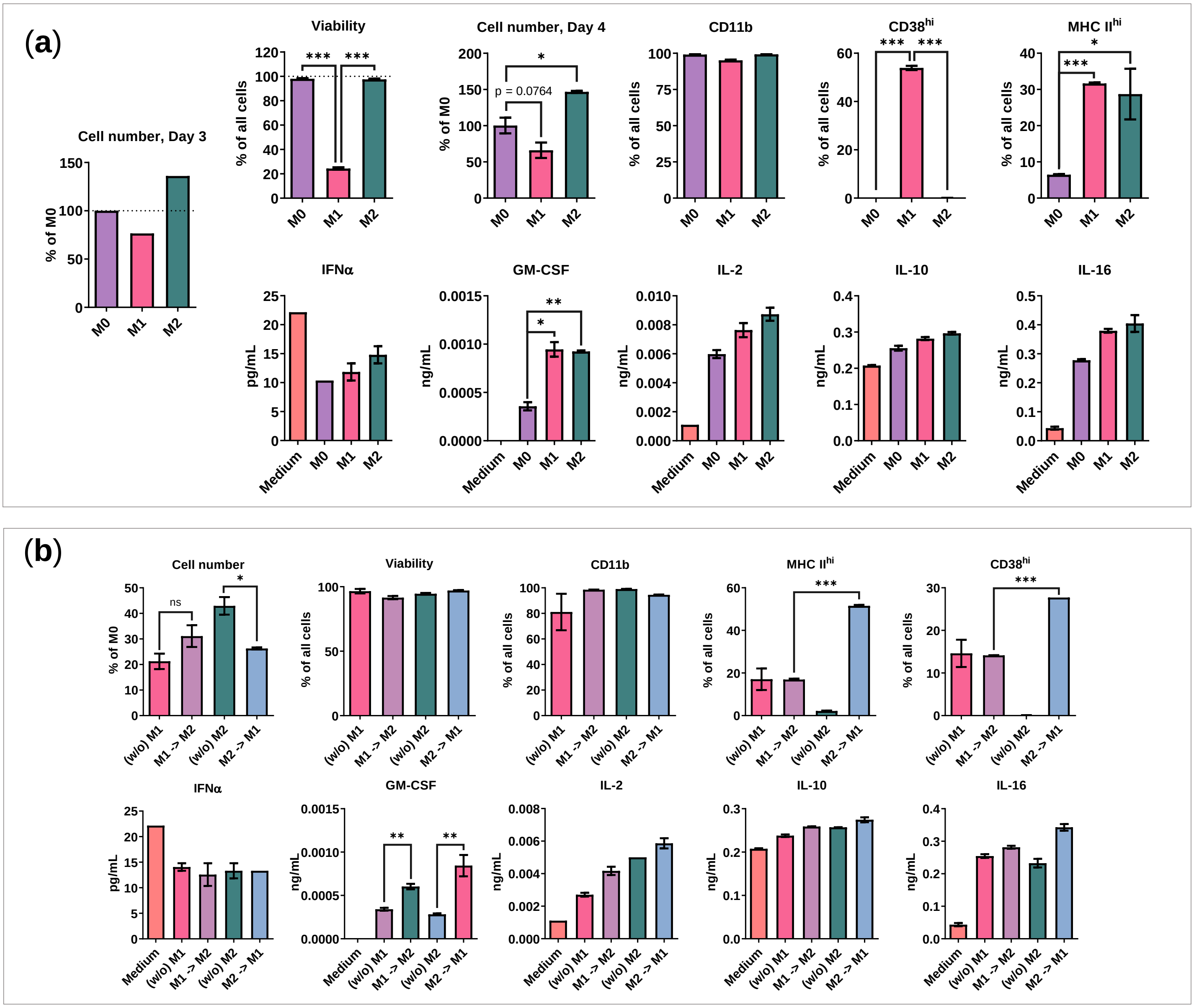


**Figure S1.** BMDMs programming and reprogramming in free-floating 3D conditions. BMDMs were plated in 96-well Black Round Bottom Ultra-Low Attachment plate in a medium containing programming factors: 50 ng/mL recombinant mouse IFN$\gamma$ and 100 ng/mL Pam3SCK4 to achieve M1 phenotype or with 20 ng/mL IL-4 to achieve M2 phenotype. (**a**) Cell number, flow cytometry determined marker expression and levels of cytokines (ELISA/Luminex) of 3D polarized macrophages 72 h after activation. Data are presented as % of all cells. (**b**) Cell number, flow cytometry determined marker expression and levels of cytokines (ELISA/Luminex) of 3D reprogrammed macrophages 72 h after reprogramming. Data are presented as % of all cells.
Data are shown as the mean of two experiments ± SEM ($n$ = 2, each experiment is a pool of 4 biological replicates).

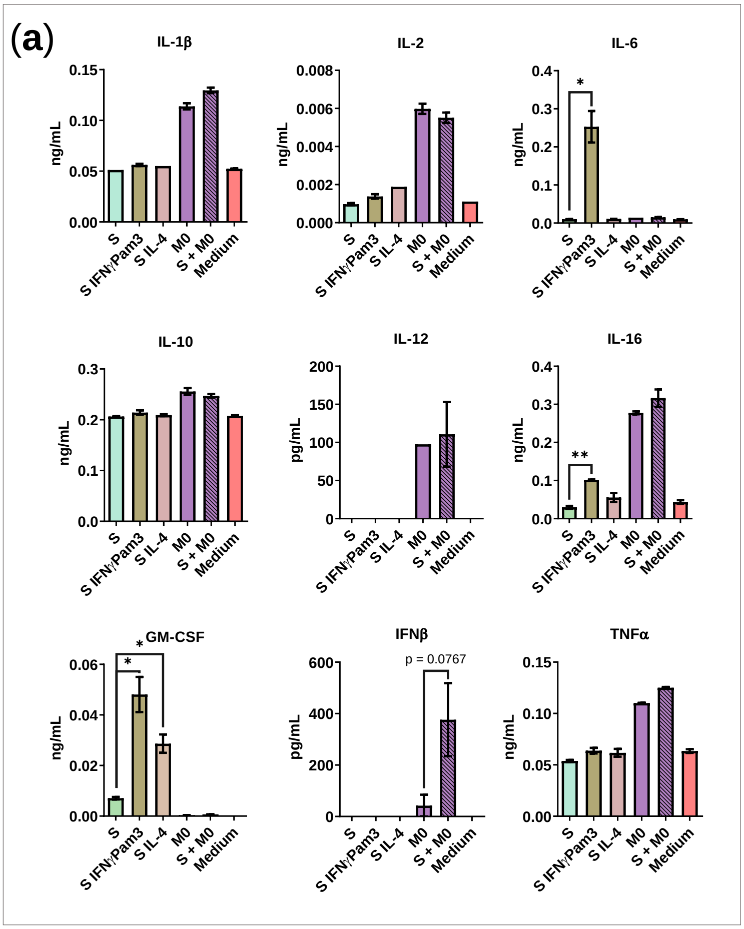


**Figure S2.** Cytokines produced by 4T1/GFP cancer cell spheroid without stimulation and upon stimulation with IL-4 or IFNγ and Pam3SCK4. Cell conditioning media from 3D monocultures and co-cultures were collected 72 h after the addition of IL-4 and IFNγ/PAM3SCK4. Then the cytokines were determined using ELISA/Luminex. Data are shown as the mean of two experiments ± SEM (*n* = 2, each experiment is a pool of 4 biological replicates).

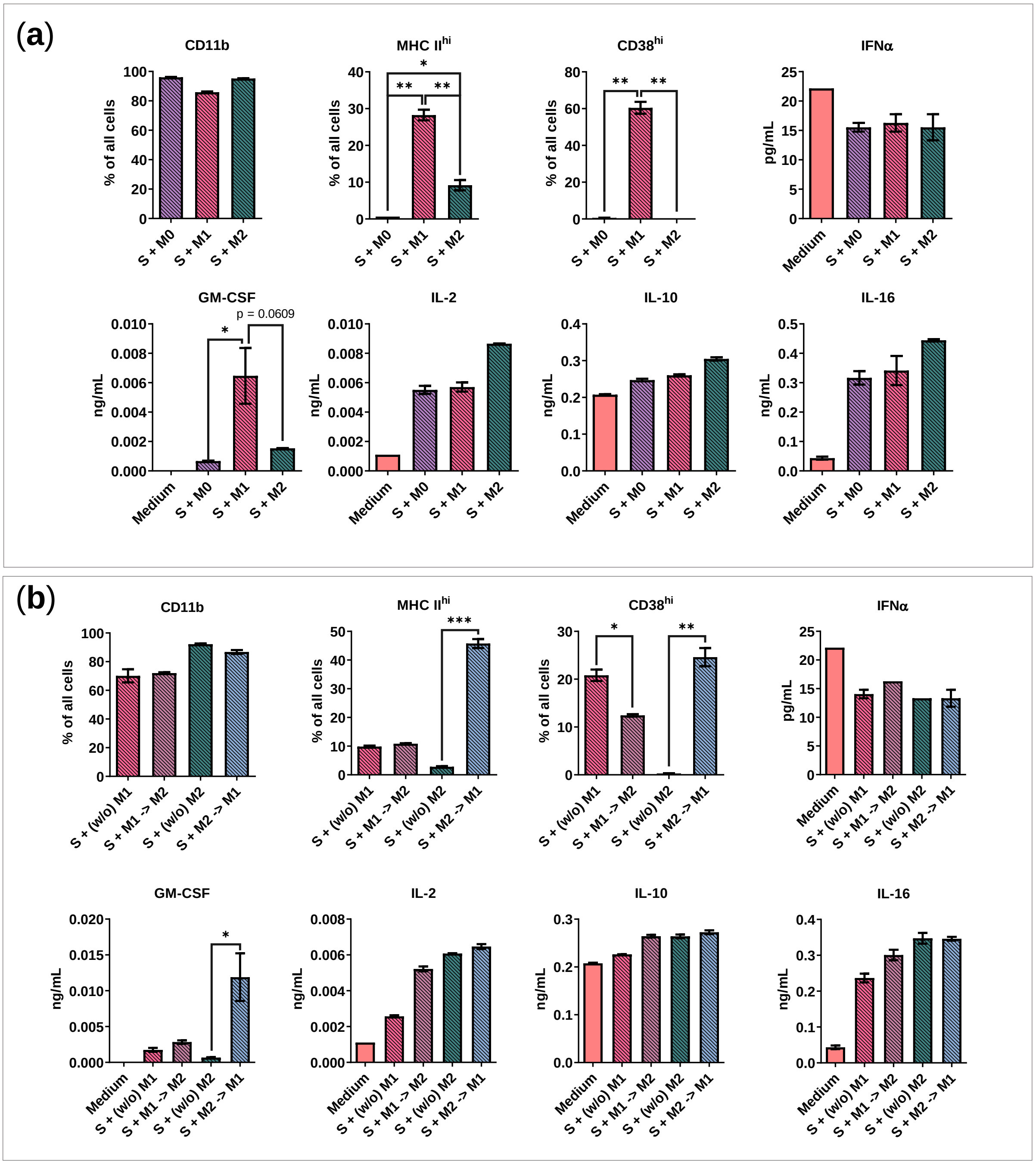


**Figure S3.** BMDMs programming and reprogramming in free-floating 3D conditions in the presence of 4T1 cancer cell spheroid. (**a**) Flow cytometry determined marker expression and levels of cytokines (ELISA/Luminex) of 3D macrophages polarized in the presence of cancer cell spheroid 72 h after activation. Data are presented as % of all cells. (**b**) Flow cytometry determined marker expression and levels of cytokines (ELISA/Luminex) of 3D macrophages reprogrammed in the presence of cancer cell spheroid 72 h after reprogramming. Data are presented as % of all cells.

Data are shown as the mean of two experiments ± SEM ($n$ = 2, each experiment is a pool of 4 biological replicates).

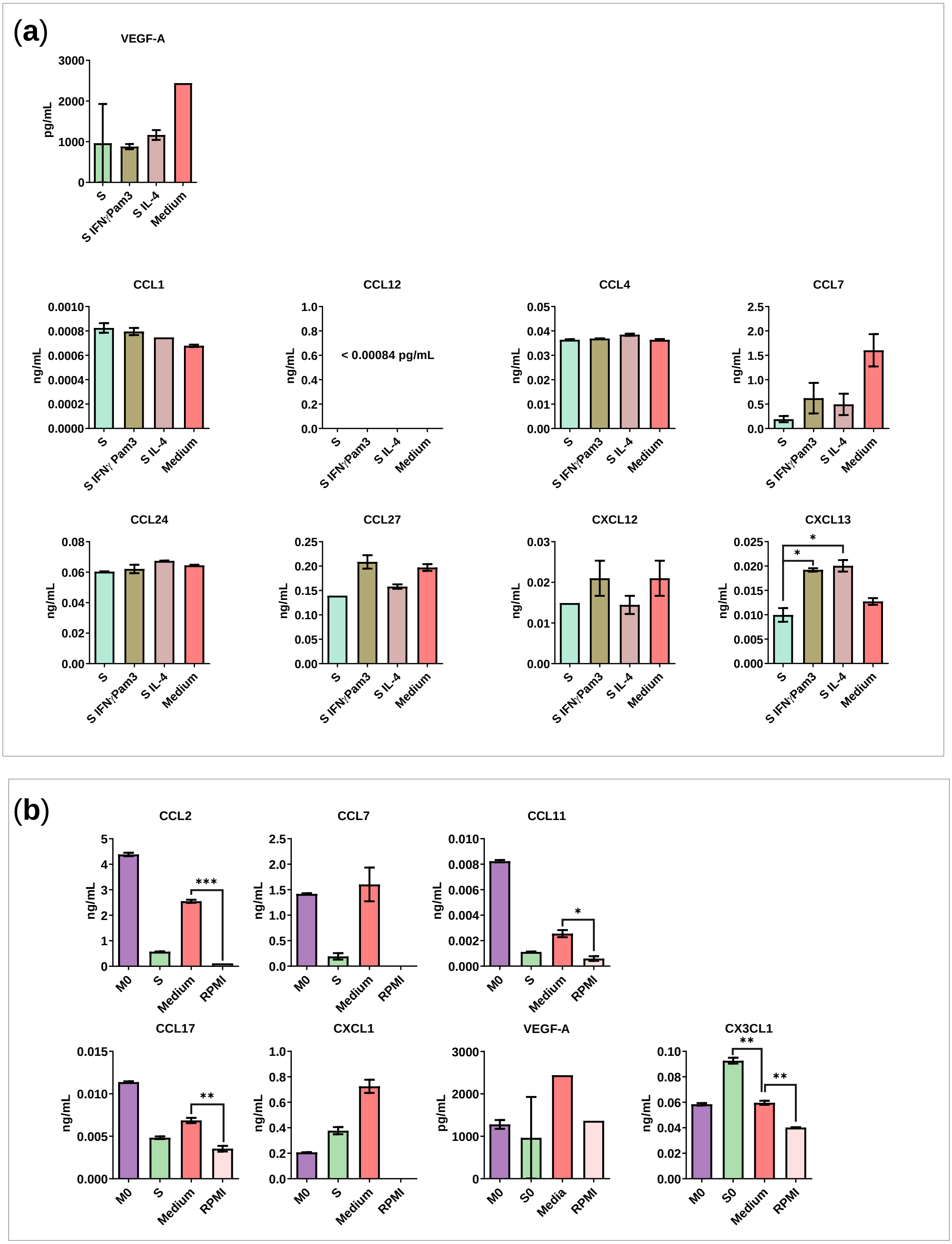


**Figure S4.** Chemokines produced by macrophages and cancer cell spheroid in 3D conditions. Cell conditioning media from monocultures and co-cultures were collected 72 h after the addition of IL-4 and IFNγ/PAM3SCK4. Then the chemokines were determined using ELISA/Luminex. (**a**) Chemokines produced by the 4T1/GFP cancer cell spheroid and upon stimulation with IL-4 or IFNγ/Pam3SCK4. (**b**) Chemokines found in the BMDM cultivation medium containing L929-CM in comparison with the medium of non-polarized macrophages (M0), cancer cell spheroid (S), and RPMI medium without L929-CM (RPMI).
Data are shown as the mean of two experiments ± SEM ($n$ = 2, each experiment is a pool of 4 biological replicates).

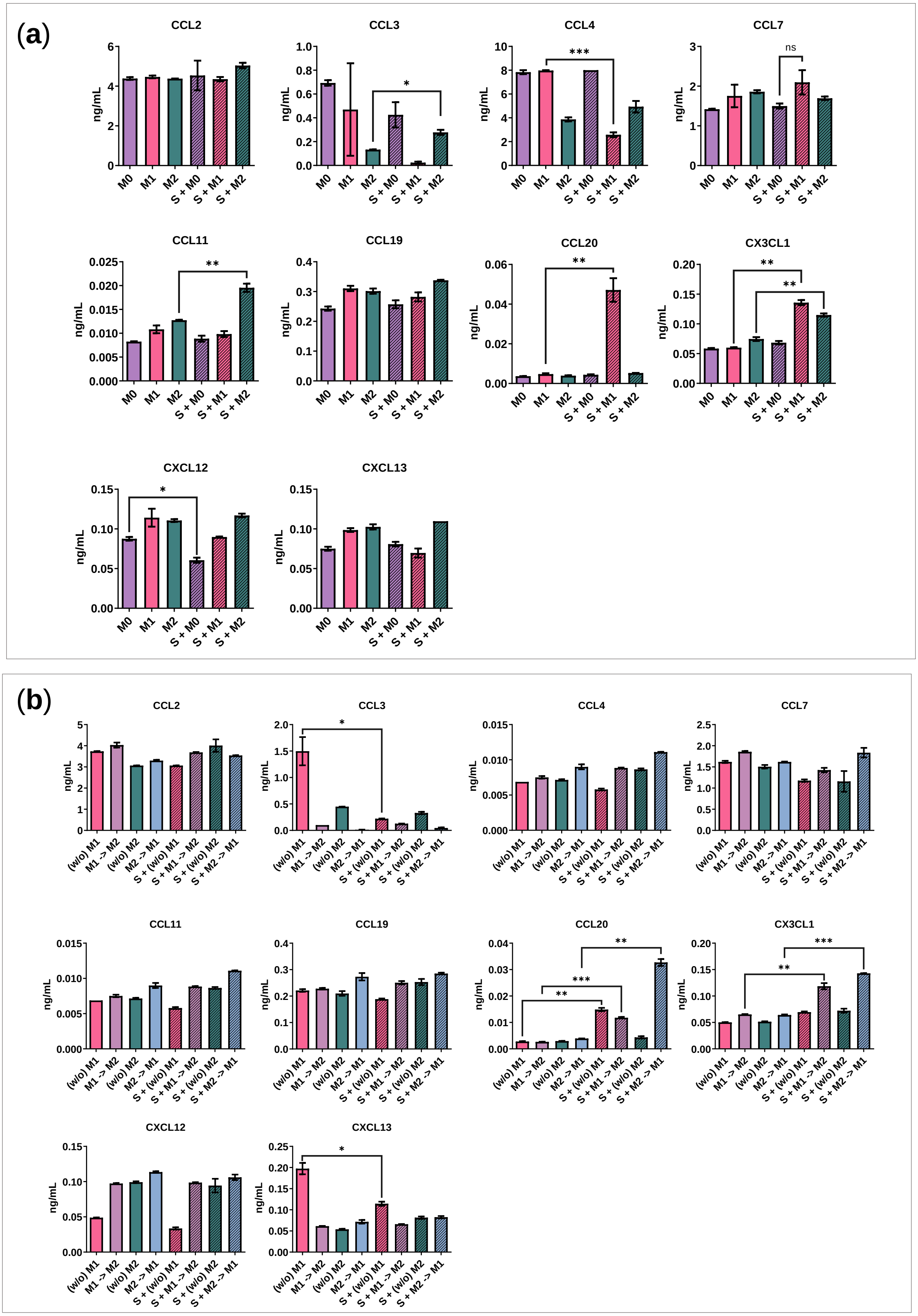


**Figure S5.** Chemokines produced by programmed/reprogrammed macrophages and cancer cell spheroid in 3D conditions. Cell conditioning media from monocultures and co-cultures were collected 72 h after the addition of IL-4 and IFN$\gamma$/PAM3SCK4. Then the chemokines were determined using ELISA/Luminex. (**a**) Chemokines found in the medium of programmed macrophages in the presence (S + M) and in the absence (M) of cancer cell spheroid. (**b**) Chemokines found in the medium of reprogrammed macrophages in the presence and in the absence of cancer cell spheroids.

Data are shown as the mean of two experiments ± SEM ($n$ = 2, each experiment is a pool of 4 biological replicates).

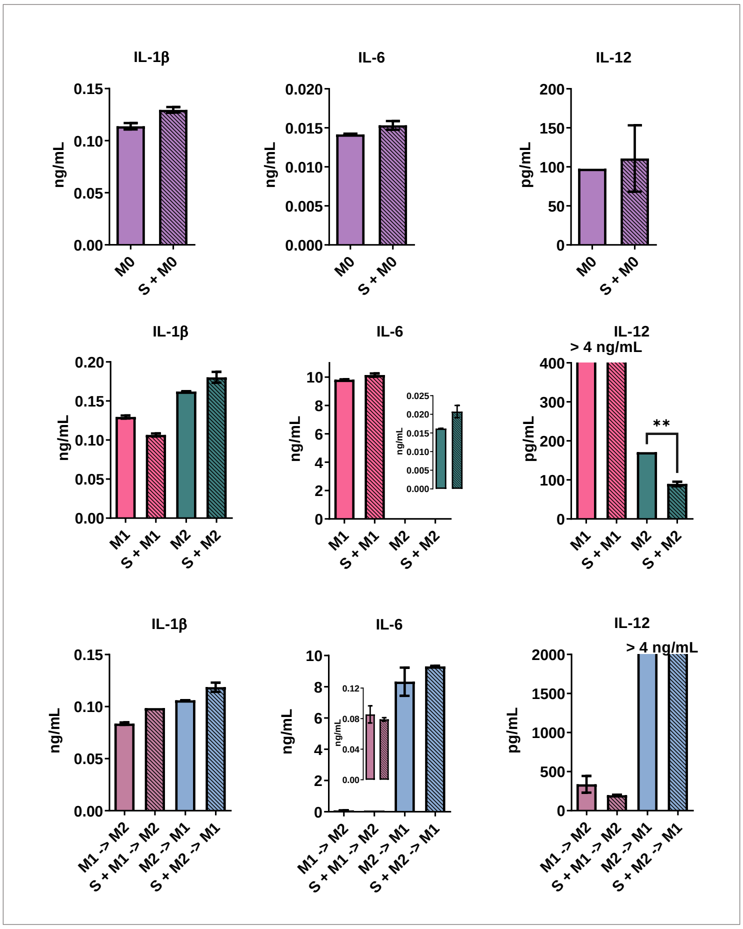


**Figure S6.** Cytokines produced by macrophages in the presence (S + M) and absence (M) of 4T1/GFP cancer cell spheroid. Cell conditioning media from 3D monocultures and co-cultures were collected 72 h after the addition of IL-4 and IFNγ/PAM3SCK4. Then the cytokines were determined using ELISA/Luminex. Data are shown as the mean of two experiments ± SEM (*n* = 2, each experiment is a pool of 4 biological replicates).

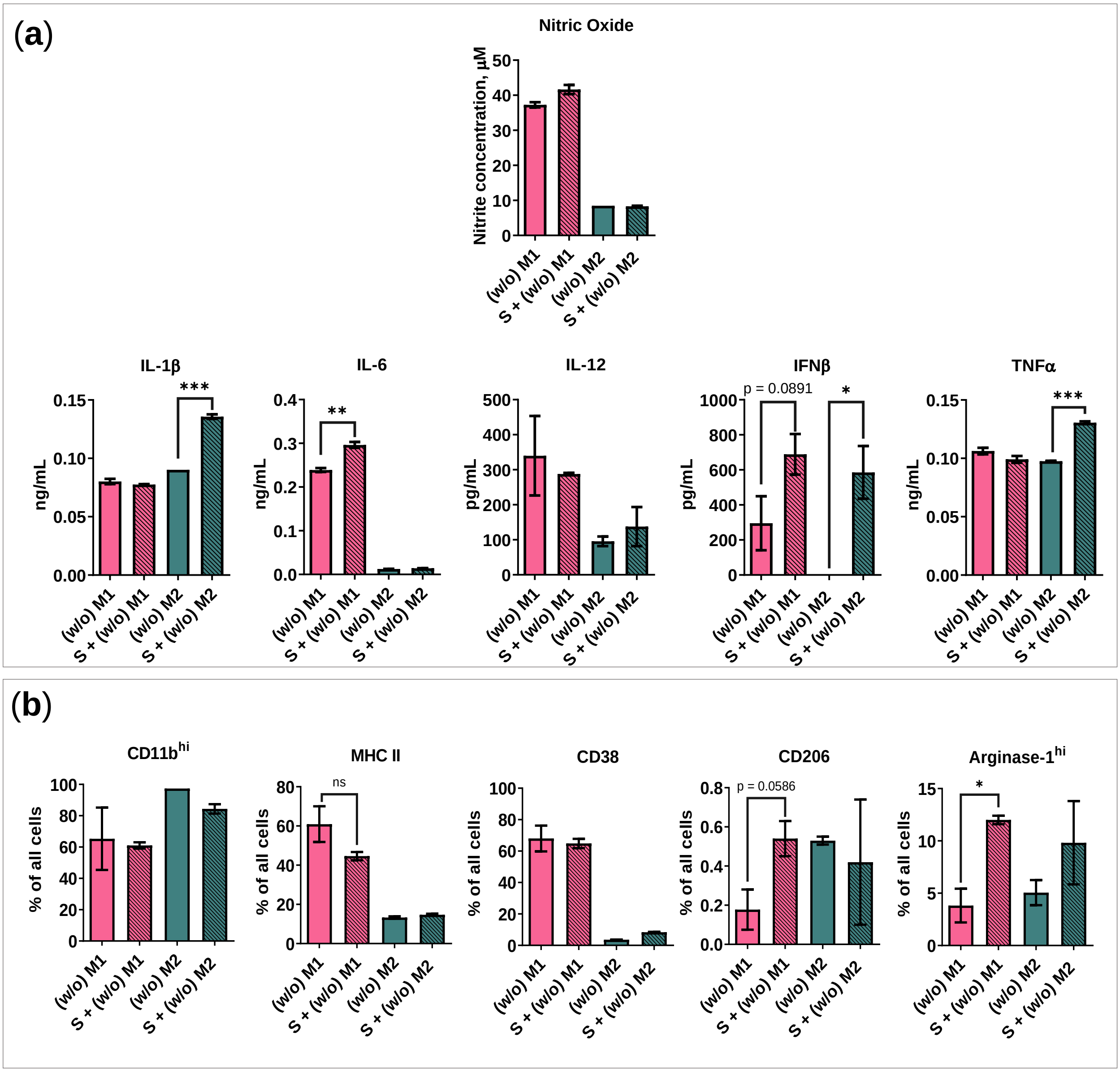


**Figure S7.** 4T1 breast cancer cell spheroid influences macrophages. Macrophages were co-cultivated with breast cancer cell spheroid 24 h before programming and 72 h after. (**a**) Changes in the levels of secreted cytokines of macrophages cultivated in the presence (S + M) and absence (M) of cancer cell spheroid. (**b**) Changes in cell surface marker levels of macrophages cultivated in the presence (S + M) and the absence (M) of cancer cell spheroid. Cells have been analyzed using flow cytometry. Data are shown as the mean of two experiments ± SEM ($n = 2$, each experiment is a pool of 4 biological replicates).
